# Do microbiota rhythms influence cognitive performance? - A narrative review

**DOI:** 10.3389/fnmol.2026.1903770

**Published:** 2026-08-04

**Authors:** Mateusz Kapela, Ewelina Barszcz, Karolina Michno, Dominika Zielińska, Dominik Strzelecki, Oliwia Gawlik-Kotelnicka

**Affiliations:** Department of Affective and Psychotic Disorders, Medical University of Lodz, Lodz, Poland

**Keywords:** chrononutrition, circadian rhythms, cognition, gut microbiota, gut-brain axis, microbial metabolites

## Abstract

The gut microbiota exhibits diurnal oscillations that are increasingly recognized as important modulators of host physiology, including brain function. Increasing evidence suggests that diurnal oscillations in microbial composition and activity may play a role in shaping cognitive performance. Microbiota and host circadian systems are tightly interconnected through feeding-fasting cycles, light-driven central clock signaling, and peripheral metabolic regulation, leading to coordinated rhythmicity across physiological systems. Disruptions to these rhythms, such as irregular eating patterns or circadian misalignment, have been associated with alterations in microbial profiles and impaired cognitive outcomes. Microbiota-derived metabolites, including short-chain fatty acids, tryptophan metabolites, and bile acids, serve as key signaling molecules that mediate communication along the gut-brain axis and influence processes such as memory, attention, and executive function. Emerging data support the existence of an integrated microbiota-circadian-cognition axis, in which microbial rhythmicity contributes to the regulation of neural and behavioral functions. Although current findings point to a meaningful association between microbiota rhythms and cognitive function, the evidence remains largely indirect and heterogeneous, showing that the time-related links between the microbiota and the brain are complex and not easy to explain. The aim of this narrative review is to synthesize current evidence on the relationships between microbiota rhythmicity, circadian regulation, and cognitive function, with a focus on possible mechanisms and potential clinical applications.

## 1 Introduction to gut microbiota and circadian rhythms

### 1.1 Gut microbiota - definition and function

The human microbiota comprises a diverse and dynamic community of microorganisms, including bacteria, viruses, and single-celled eukaryotes, that colonize various anatomical sites of the body. Among these, the gastrointestinal tract represents the most densely populated and metabolically active environment. The gut microbiota exists in a symbiotic relationship with the host and plays a fundamental role in maintaining physiological homeostasis (Thursby and Juge, 2017). One of the key functions of the gut microbiota is its metabolic activity, which complements host physiology by providing enzymatic capabilities not encoded in the human genome. This includes the breakdown of complex dietary components, such as polysaccharides and polyphenols, as well as the synthesis of essential vitamins and bioactive compounds. Through these processes, microbiota generate a wide range of metabolites that influence systemic biological functions (Rowland et al., 2018; Adak and Khan, 2019).

### 1.2 Overview of the gut-brain axis

The gut microbiota is a central component of the gut-brain axis, a bidirectional communication network that links the gastrointestinal tract and the central nervous system. This communication occurs through neural, endocrine, and immune pathways – including activation of the vagus nerve, systemic circulation of microbial metabolites such as short-chain fatty acids (SCFAs), and modulation of immune signaling – enabling microbial activity to influence host neurophysiology at multiple levels. Among the key signaling molecules involved are gamma-aminobutyric acid (GABA) and tryptophan-derived metabolites (e.g., serotonin), which contribute to the regulation of both the peripheral and central nervous systems (O’Riordan et al., 2025).

In addition to their signaling roles, these metabolites act as important immune modulators. SCFAs regulate cytokine production and microglial activation, while serotonin and GABA influence immune cell activity. Through these integrated pathways, the gut microbiota influences several domains of brain function, including learning, memory, attention, executive function, and emotional regulation. These cognitive processes depend on intact neuronal signaling, neuroplasticity, and neuroimmune homeostasis, all of which are shaped by microbial-derived metabolites and immune mediators (O’Riordan et al., 2025).

Under physiological conditions, the host and microbiota maintain a balanced state referred to as eubiosis. Disruptions in microbial composition or function, known as dysbiosis, may lead to increased intestinal permeability and systemic immune activation. This, in turn, promotes neuroinflammatory processes that negatively affect neuronal communication, synaptic plasticity, and alter neurotransmitter availability, consequently creating conditions that are unfavorable for optimal cognitive function. Such disruptions also interact with the host’s circadian system, as neuroinflammation can alter clock gene expression and sleep architecture, which may further compromise multiple domains of cognitive performance (Zhang et al., 2025).

### 1.3 Circadian rhythms - basic concepts

Circadian rhythms are a fundamental feature of human physiology, reflecting the temporal organization of biological processes. They are endogenous oscillations with an approximately 24-h cycle that coordinate functions such as the sleep-wake cycle, metabolism, hormone secretion, and body temperature in response to predictable environmental changes. The circadian system in mammals consists of several interdependent structures. The master circadian clock is located in the suprachiasmatic nucleus (SCN) of the hypothalamus, which coordinates peripheral clocks across tissues and thereby regulates both behavioral and humoral rhythms. Due to its location, the SCN receives light input via retinal ganglion cells, which transmit light-derived signals that serve as the primary synchronizing cue for the central clock. In turn, peripheral clocks are regulated by multiple endogenous factors from the SCN, including autonomic innervation, endocrine signaling, temperature, and local signals. At the cellular level, these rhythms are modulated through a highly complex network of internal transcriptional-translational mechanisms, which maintain their oscillatory nature and ensure their precise temporal functioning (Xie et al., 2019).

Each individual exhibits a distinct chronotype, reflecting their preferred timing of sleep and activity. Chronotype is influenced by numerous factors, including age, sex, shift work, and altered activity and rest patterns. Notably, polymorphisms in core circadian clock genes have been associated with inter-individual and inter-ethnic differences in chronotype, suggesting a genetic contribution to human variability in circadian phase preference (Smith et al., 2009). In this context, a state in which circadian rhythms lose proper synchronization with each other and environmental cycles, leading to disrupted biological processes, is defined as chronodisruption (Erren and Reiter, 2009). Chronodisruption has been implicated in a range of medical conditions and represents an important risk factor for neurological and metabolic dysfunction (Montaruli et al., 2021). Recent evidence indicates that the gut microbiota also exhibits circadian dynamics, with daily fluctuations in both microbial composition and metabolic activity. These rhythms are closely linked to the host’s internal biological clock and are modulated by environmental factors such as feeding patterns and light-dark cycles (Li J. et al., 2025).

Cognitive performance is therefore a particularly relevant focus, as it is sensitive to circadian timing and may reflect the functional consequences of microbiota-related signaling within the gut–brain axis (Chellappa et al., 2018; Zhang et al., 2025). Taken together, gut microbiota and circadian rhythms represent two fundamental regulatory systems that shape host physiology across multiple levels. Although traditionally studied separately, growing evidence suggests that these systems are closely interconnected. Understanding their interaction provides the conceptual framework for exploring how temporal organization and microbial activity jointly influence brain function and shape cognitive processes across the lifespan.

The present narrative review aims to synthesize current evidence on the bidirectional interactions between gut microbiota, circadian rhythms, and cognitive function, with a focus on the underlying mechanisms and their potential practical implications for cognitive health.

## 2 Materials and methods

A search of the scientific literature was performed using PubMed and Web of Science, encompassing publications from 2000 to 2026. Search terms included combinations of keywords related to gut microbiota, microbiota-derived metabolites, cognitive function, and circadian rhythms, supplemented by terms associated with neurophysiological regulation, sleep, and microbiota–gut–brain axis signaling. The initial search yielded approximately 3,526 records. Of these, 1,132 were excluded at the database level due to unavailability of full text, leaving 2,394 records for further screening. Following the removal of duplicates, titles and abstracts were screened against the predefined eligibility criteria. A total of 121 publications were ultimately included in this narrative review.

Studies were considered eligible if they examined the relationship between gut microbiota and circadian rhythms and/or cognitive function, and reported on at least one of the following outcomes: gut microbiota composition or activity, circadian rhythm parameters (e.g., sleep, chronotype, circadian alignment), or cognitive outcomes including memory, attention, and executive function. Both preclinical and clinical study designs were included – comprising observational studies, randomized controlled trials, interventional studies, and relevant systematic reviews and meta-analyses. Articles were excluded if they were not published in English, lacked original data, or did not directly address the microbiota–circadian–cognition axis.

## 3 How does microbiota impact circadian rhythms and vice versa?

### 3.1 Circadian rhythms of gut microbiota

Emerging studies indicate that the gut microbiota has a circadian rhythm of its own. Zarrinpar et al. (2014) found that in mice, cyclical changes in the gut microbiome affected all major phyla. During nocturnal feeding, the proportion of Firmicutes species peaked, and bottomed out during daytime fasting. Conversely, Bacteroidetes and Verrucomicrobia species peaked during daytime fasting and decreased during nocturnal feeding. These cyclical fluctuations were accompanied by changes in the diversity of the microflora environment, rising with feeding and falling with fasting (Zarrinpar et al., 2014). These findings suggest that the microbiota’s circadian rhythm might be dependent on the consumption time. Moreover, not only does microbiota composition undergo cyclic fluctuations, but also its functions. Thaiss et al. (2014) uncovered that in mice, during the dark phase, functions involved in energy metabolism, DNA repair, and cell growth were performed favorably, whereas in the light phase, pathways involved in detoxification, motility, and environmental sensing were more prevalent. Importantly, the host circadian clock was found to be essential for generating diurnal oscillations in the gut microbiota, primarily through the regulation of feeding rhythms. Circadian disruption, such as chronic jet lag, led to altered feeding behavior, loss of microbial rhythmicity, and dysbiosis, which in turn contributed to metabolic dysfunction (Thaiss et al., 2014). These findings indicate that microbial oscillations are governed primarily by time of consumption rather than the light–dark cycle in particular. Consistently, SCFAs - metabolites produced by microbiota, also exhibit clear circadian oscillations in the gut, with concentrations of key metabolites such as acetate and butyrate showing day–night fluctuations that are linked to mice feeding cycles (Tahara et al., 2018). The observed temporal fluctuations in microbial composition and metabolite production point to a complex regulatory network underlying gut microbial rhythmicity. Several factors contribute to the regulation of gut microbial rhythmicity, including nutritional and environmental factors, host circadian regulation, and physiological state (Wang H. et al., 2022). Although research in this area is mostly based on animal studies, there is also evidence in human-based studies. In humans, analysis of 1,943 subjects showed diurnal fluctuations in microbiota composition, with higher Bacteroidetes abundance at night and higher Firmicutes during the day (Reitmeier et al., 2020). Collectively, these findings indicate that gut microbial composition and function exhibit diurnal variation shaped by feeding rhythms and host circadian regulation. However, most mechanistic evidence comes from animal models, and the physiological significance of microbial rhythmicity in humans remains to be clarified.

### 3.2 How do circadian rhythms and microbiota influence each other?

The relationship between circadian rhythms and gut microbiota is bidirectional and spans across developmental stages. In animal models, acute sleep deprivation disrupts circadian rhythms in mice and is accompanied by gut microbiota dysbiosis, reduced microbial diversity, impaired production of SCFAs, and, therefore, inflammation and gut barrier dysfunction (Yang et al., 2023).

The directionality of the relationship between the circadian rhythm and gut microbiota remains poorly understood. Gut microbiota was proposed as a regulator of hypothalamic–pituitary–adrenal (HPA) axis rhythmicity. Microbial repletion disrupted brain circadian signaling and glucocorticoid oscillations, while microbiota transplantation of *Lactobacillus reuteri* partially restored normal temporal patterns of stress hormone release, highlighting a causal role of microbes in circadian stress regulation (Tofani et al., 2025). Similarly, sleep desynchronization in mice was associated with impaired HPA axis regulation, along with disrupted microbial diversity, reduced Bacteroidota, and increased Firmicutes, which led to dysbiosis (Wu et al., 2025). Thus, these studies highlight the potential connection between the HPA-axis, gut microbiota, and disruption of the circadian rhythm. However, this evidence is based on animal models of studies and needs confirmation in human trials.

Moreover, recent evidence has highlighted melatonin and immune signaling as additional mediators linking circadian rhythms with gut microbiota dynamics. Melatonin appears to act as a central mediator in the bidirectional communication between the host circadian system and gut microbiota. *Enterobacter aerogenes* has been shown to exhibit circadian patterns in motility and swarming that are enhanced by physiological levels of melatonin, suggesting that bacterial rhythmicity can respond directly to host-derived circadian signals (Paulose et al., 2016). In mice, chronic sleep restriction led to a loss of circadian rhythm in the core and peripheral families of the colonic microbiota, an effect that was partially restored by melatonin supplementation, particularly within Muribaculaceae and Lachnospiraceae (Li et al., 2023).

In addition to melatonin-mediated effects, immune pathways represent another key mechanism linking circadian rhythms and gut microbiota. The immune system is closely linked to sleep, and its activation, which could be triggered by dysbiosis, affects sleep regulation (Besedovsky et al., 2019). The relationship between inflammatory mediators such as IL-6 and microbiota appears context-dependent: while elevated nocturnal IL-6 has been associated with worse sleep quality in clinical populations, greater microbiome diversity has been linked to better sleep alongside higher IL-6 levels in other cohorts, suggesting that IL-6 may exert differential roles depending on the physiological state of the organism (Burgos et al., 2006; Smith et al., 2019).

Interventions targeting the gut microbiota further support its role in circadian regulation. In a study by Leone et al. (2022), germ-free mice displayed altered circadian rhythmicity, which was partially restored through microbial transplantation from conventionally raised mice, suggesting that gut microbes modulate host circadian rhythms. Importantly, microbiota metabolites, such as SCFAs, also influence the relationship between circadian rhythms and gut microbiota. For example, *in vitro* studies reported that microbial metabolites, particularly SCFAs, can shift and synchronize intestinal circadian clocks through histone deacetylase 3-dependent epigenetic mechanisms, indicating that gut microbiota can regulate host biological timing (Fawad et al., 2022).

**FIGURE 1:**
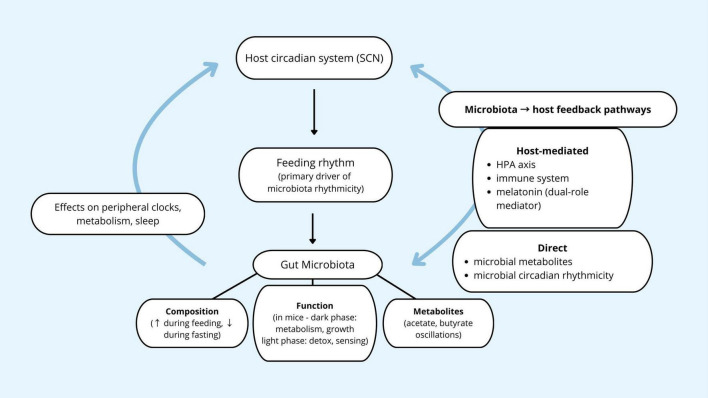
Mechanisms linking host circadian rhythms and gut microbiota: a bidirectional model. ↑ *increased*; ↓ decreased; HPA, hypothalamic-pituitary-adrenal; SCN, suprachiasmatic nucleus.

Taken together, current evidence supports a bidirectional relationship between host circadian regulation and the gut microbiota, potentially mediated by feeding patterns, HPA-axis activity, melatonin, immune signaling, and microbial metabolites. Nevertheless, the direction and relative contribution of these pathways remain uncertain in humans, as much of the mechanistic evidence comes from animal and *in vitro* studies. These interactions are summarized in Figure 1.

### 3.3 Circadian–microbiota interactions in humans

Human studies provide converging support for a bidirectional relationship between circadian rhythms and gut microbiota, though direct causal evidence remains limited.

Observational data across developmental stages show that microbiota diversity is positively associated with sleep efficiency and total sleep time in adults (Smith et al., 2019), while in infants and preschool-aged children, sleep duration and efficiency correlate with specific microbial taxa including *Bifidobacterium* (Schoch et al., 2022; Wang H. et al., 2022). In patients with insomnia, meta-analytic data demonstrate reduced alpha-diversity and distinct beta-diversity profiles compared to healthy controls, alongside shifted Firmicutes-to-Bacteroidetes ratios (Chen et al., 2026). In patients with obstructive sleep apnea/hypopnea syndrome (OSAHS), gut microbiome alterations were associated with a reduction in beneficial bacteria and an increase in lipopolysaccharide (LPS)-producing taxa. Similar microbiota alterations – including reduced alpha-diversity and increased abundance of pro-inflammatory genera such as *Escherichia/Shigella*, *Blautia*, and *Dialister* – have been consistently reported in night-shift workers (Grasa-Ciria et al., 2025). One study found no significant microbiota changes following short-term sleep restriction, though its interpretation is limited by a small sample size (*n* = 11) (Zhang et al., 2017).

Intervention-based evidence further supports the relevance of microbiota modulation for circadian-related outcomes. A meta-analysis demonstrated significant improvements in sleep quality following daily consumption of *Lactobacillus gasseri* CP2305 in adults with mild to moderate stress (Chu et al., 2023), while supplementation with *Bifidobacterium animalis* subsp. lactis BLa80 reduced sleep onset latency and daytime fatigue without improving overall sleep quality (Liu et al., 2025). Fecal microbiota transplantation has shown preliminary promise in chronic insomnia, with one real-world study reporting significant reductions in insomnia severity index scores alongside shifts in microbiota composition (Fang et al., 2023).

Taken together, human data support an association between circadian disruption and microbiota alterations, and suggest that microbiota-targeted interventions may influence sleep-related outcomes. However, the causal direction of these relationships and the underlying mechanisms remain to be established in adequately powered, longitudinal human trials.

## 4 Microbial metabolites in circadian microbiota–host signaling and cognitive function.

Microbiota-derived metabolites represent an important link between the diurnal metabolic activity of the gut microbiota and host physiology, enabling signal transmission both locally within the intestine and systemically after entering the circulation (Wikoff et al., 2009; Thaiss et al., 2016). In relation to cognitive function, the strongest evidence currently concerns pathways involving short-chain fatty acids and tryptophan metabolism, whereas for bile acids, the available data more strongly support their role in circadian regulation than in the direct modulation of cognitive processes (Wang X. et al., 2023; Bello et al., 2024; Song et al., 2025; Liao et al., 2026).

### 4.1 Short-chain fatty acids (SCFAs)

Short-chain fatty acids (SCFAs), particularly acetate, propionate, and butyrate, are among the best-characterized microbiota-derived metabolites and have been implicated in circadian regulation as well as brain-related processes (Tahara et al., 2018; Shi et al., 2021; Fawad et al., 2022). They are produced by bacterial fermentation of non-digestible dietary substrates, and their generation depends on substrate availability, diet, and the presence of SCFA-producing taxa (Tahara et al., 2018; Van-Wehle and Vital, 2024; Kirschner et al., 2025).

Short-chain fatty acids may also influence circadian regulation, as gut microbe-generated SCFAs entrain intestinal epithelial rhythms through a histone deacetylase inhibition-dependent mechanism in organoid and mouse models (Fawad et al., 2022). Their circadian role is further supported by day-night variation in caecal SCFAs concentrations in mice, time-dependent shifts of peripheral clocks after SCFAs administration, and rhythmic effects on colon contractility (Tahara et al., 2018; Segers et al., 2019). In humans, diurnal plasma SCFAs oscillations were reported to be disrupted in shift workers consuming alcohol (Swanson et al., 2020). In a sleep-deprivation model, lower colonic butyrate was accompanied by memory impairment, whereas butyrate administration ameliorated inflammatory responses and memory deficits, supporting the view that disrupted SCFAs signaling may contribute to the cognitive consequences of sleep loss (Wang X. et al., 2023).

Evidence for cognitive effects is the strongest in animal models. Long-term fiber deprivation reduced SCFAs’ availability and was accompanied by memory impairment, hippocampal microglial activation, synaptic alterations, and increased gut permeability (Church et al., 2023). By contrast, a high-pectin diet was associated with reduced hippocampal neuroinflammation, increased brain-derived neurotrophic factor (BDNF), and acetate-mediated effects on BDNF (Church et al., 2023).

Human evidence remains largely observational. Observational studies have linked higher dietary butyrate intake to better cognition in older adults, while altered circulating SCFAs profiles have been associated with cognitive impairment in HIV-associated neurocognitive disorder and Alzheimer-related cognitive impairment (Chen et al., 2025; Marizzoni et al., 2025; Tu et al., 2025).

### 4.2 Tryptophan metabolites

Tryptophan metabolism represents an important microbiota-gut-brain signaling axis because dietary tryptophan is partitioned across host serotonin- and kynurenine-related pathways and microbiota-derived indole pathways (Renga et al., 2023). Gut microbes contribute directly by generating biologically active tryptophan derivatives, including metabolites with aryl hydrocarbon receptor activity, and microbially produced tryptophan metabolites can improve intestinal barrier integrity through this pathway (Dong et al., 2020; Scott et al., 2020). This is relevant to gut-brain communication because indole, indole-3-acetate, indole-3-propionate, and tryptamine modulated both gut and blood-brain barrier models in a dose- and cell-type-dependent manner (Rosell-Cardona et al., 2025).

Its circadian relevance is supported more directly by evidence that tryptophan-metabolizing bacteria and host intestinal tryptophan-metabolizing enzymes display diurnal rhythmicity, and that microbial depletion alters rhythms in gut barrier function and host tryptophan metabolism (Gheorghe et al., 2024). This temporal dimension is further supported by findings that the gut microbiota regulates host melatonin production through epithelial MyD88, whereas bacterial metabolites derived from tryptophan and phenylalanine were shown to induce melatonin synthesis and extend sleep duration in mice (Lee et al., 2024; Liu et al., 2024). In a mouse model of circadian disruption, fecal microbiota from affected mice reproduced cognitive deficits. Reduced tryptophan and 5-hydroxytryptophan (5-HTP) levels were also observed, and improvement was noted following dietary tryptophan supplementation (Song et al., 2025). Consistently, irregular light-dark exposure impaired recognition and spatial memory together with dysregulation of the tryptophan-5-HT pathway, and recognition memory improved after 5-HTP supplementation (Liao et al., 2026).

Evidence for brain-related effects comes mainly from experimental models. Indole produced by tryptophanase-expressing microbes exerted neurogenic effects in the adult mouse hippocampus via the aryl hydrocarbon receptor, and recent work has identified indole-3-propionic acid (IPA) as a plausible candidate metabolite in mouse models of autism spectrum disorder and Alzheimer’s disease (Wei et al., 2021; Wang X. et al., 2023; Jiang et al., 2024). In these models, IPA was linked to abnormal microglial synaptic pruning and to the restoration of social function, hippocampal inhibitory synaptic transmission, and cognition; in the Alzheimer’s disease model, some of these effects were reproduced by IPA supplementation or by colonization with the IPA-producing bacterium *Clostridium sporogenes* (Wang X. et al., 2023; Jiang et al., 2024; Li J. et al., 2025). Human evidence remains more limited and largely associative, although gut microbial tryptophan metabolites have been linked with brain activity and symptom severity in autism spectrum disorder (Aziz-Zadeh et al., 2025).

### 4.3 Bile acids

Bile acids form a distinct class of microbiota-modified metabolites because gut bacteria reshape the bile acid pool through reactions such as 7α-dehydroxylation, a key step in the generation of secondary bile acids (Funabashi et al., 2020; Kim et al., 2022). Beyond their role in lipid digestion, bile acids also function as signaling molecules that engage host receptor pathways with systemic metabolic and signaling consequences, notably through farnesoid X receptor (FXR)- and Takeda G protein-coupled receptor 5 (TGR5)-related signaling (Pathak et al., 2018).

Their circadian relevance is further supported by mouse studies showing that intestinal epithelial clocks generate rhythms in microbial metabolic products, particularly SCFAs and bile acids, and that circadian disruption can alter the rhythmicity of intestinal bile acid metabolism (Chen et al., 2022; Heddes et al., 2022). Consistently, in healthy men, circulating bile acids showed clear 24-h variation, and this temporal organization was disrupted by sleep deprivation, but direct links to cognitive performance remain lacking (Bello et al., 2024).

In humans, chronic insomnia was associated with specific gut microbial and bile acid signatures, although the causal direction of these relationships remained unresolved. Direct evidence linking bile acids to cognition remains limited and comes mainly from disease-related settings. In Alzheimer cohorts, altered serum bile acid profiles were associated with cognitive impairment, and conjugated bile acid features correlated with cognitive status, clinical stage, and amyloid-β deposition (MahmoudianDehkordi et al., 2019; Chen et al., 2024). In aging, increased intestinal absorption of conjugated primary bile acids and ammonia was linked to cognitive decline, whereas in major depressive disorder, abnormal bile acid metabolism was associated with several dimensions of cognitive dysfunction (Jia et al., 2024; Ren et al., 2024). Further mechanistic support comes from a mouse model of cognitive dysfunction induced by androgen deprivation therapy, in which taurodeoxycholic acid supplementation improved cognitive performance and was associated with increased hippocampal TGR5 and p- extracellular signal-regulated kinases 1 and 2 (ERK1/2) signaling (Yang et al., 2025).

### 4.4 Other relevant microbial metabolites

Beyond SCFAs, tryptophan metabolites, and bile acids, several other microbial or microbiota-dependent metabolites have been proposed as additional contributors to microbiota-brain communication, although the evidence remains limited and heterogeneous. Among the candidates most directly supported by recent original studies are trimethylamine N-oxide (TMAO), phenylacetylglutamine (PAGln), gamma-aminobutyric acid (GABA)-related microbial signaling, and selected phenolic metabolites.

Human findings for TMAO are inconsistent. In middle-aged and older adults, plasma TMAO was inversely associated with memory and fluid cognition, whereas in the population-based Rotterdam Study, it was not associated overall with cognition, neuroimaging markers, or incident dementia (Brunt et al., 2021; Yaqub et al., 2024).

Experimental findings nevertheless support the biological plausibility of TMAO-related effects. Chronic TMAO exposure worsened memory-related performance in mice and aggravated synaptic plasticity deficits in a rat model of vascular dementia, while higher plasma TMAO was also reported in mild cognitive impairment, together with TMAO-related cognitive and synaptic alterations in rat models (Brunt et al., 2021; Deng et al., 2022; Shi et al., 2025).

Phenylacetylglutamine represents another microbiota-dependent candidate. It is generated through microbial conversion of dietary phenylalanine to phenylacetic acid, followed by host conjugation, and higher plasma PAGln was prospectively associated with post-stroke cognitive impairment at 3 months (Nemet et al., 2020; Fei et al., 2025).

Gamma-aminobutyric acid-related microbial signaling is another relevant pathway. In mice, *Bifidobacterium bifidum* TMC3115 increased intestinal GABA and reduced anxiety-related behavior, whereas a placebo-controlled human cross-over study of GABA-producing lactobacilli reduced cognitive reactivity to sad mood but did not improve objective cognitive performance (Casertano et al., 2024; Ikegami et al., 2024).

Selected phenolic metabolites may also be relevant. In older adults, a higher urinary microbial phenolic metabolite score was associated with better frontal cognitive performance, while in aged mice, urolithin A suppressed age-related memory impairment and reduced inflammation (Domínguez-López et al., 2024; Kubota et al., 2024).

For some of the additional metabolites discussed above, a temporal component has also been reported, although the evidence remains limited and uneven (Du et al., 2024; Tian et al., 2024; Zhu et al., 2024). In mice, chronic sleep restriction increased circulating and brain TMAO and was accompanied by impaired novel object recognition and Y-maze performance, while exogenous TMAO further worsened these deficits (Zhu et al., 2024). In another sleep-deprivation model, *Bifidobacterium breve* CCFM1025 improved novel object recognition and attenuated circadian disturbance together with shifts in gut GABA and other microbial metabolites, consistent with a contribution of neuroactive microbial signaling to the cognitive effects of sleep loss (Tian et al., 2024). For phenolic metabolites, urolithin A protected against sleep-deprivation-induced cognitive impairment in mice, while separate work showed that it modulated BMAL1 and PER2 rhythmicity in intestinal epithelial cells and attenuated inflammation-related circadian dysregulation (Du et al., 2024; Tian et al., 2024). By contrast, direct evidence linking phenylacetylglutamine rhythmicity to cognitive outcomes remains limited.

### 4.5 Human evidence on microbiota-derived metabolites, sleep, and cognitive function

Human evidence remains limited and is derived predominantly from observational studies. Higher dietary butyrate intake has been associated with better cognitive function in older adults, whereas altered circulating SCFA profiles have been reported in HIV-associated neurocognitive disorder and Alzheimer-related cognitive impairment (Tu et al., 2025; Chen et al., 2025; Marizzoni et al., 2025). Microbial tryptophan metabolites have also been associated with brain activity and symptom severity in autism spectrum disorder (Aziz-Zadeh et al., 2025).

Altered bile acid profiles have been associated with cognitive impairment in Alzheimer’s disease, cognitive decline during aging, and cognitive dysfunction in major depressive disorder (MahmoudianDehkordi et al., 2019; Chen et al., 2024; Jia et al., 2024; Ren et al., 2024). Shift work and sleep deprivation have additionally been associated with disrupted circulating SCFA and bile acid rhythms, respectively, while chronic insomnia has been linked to specific gut microbial and bile acid signatures (Swanson et al., 2020; Bello et al., 2024; Jiang et al., 2022).

Evidence concerning other metabolites is less consistent. Higher TMAO concentrations have been associated with poorer cognitive performance in some cohorts, whereas the Rotterdam Study found no overall association with cognition or incident dementia (Brunt et al., 2021; Yaqub et al., 2024). Higher PAGln levels have been prospectively associated with post-stroke cognitive impairment, while a placebo-controlled cross-over study of GABA-producing lactobacilli reduced cognitive reactivity to sad mood but did not improve objective cognitive performance (Fei et al., 2025; Casertano et al., 2024). Microbiota-derived metabolites may connect circadian microbial activity with host physiology, sleep regulation, and cognitive function. The strongest evidence concerns SCFAs and tryptophan metabolites, although most findings related to cognition derive from experimental models or observational human studies. Bile acids appear to be more consistently involved in circadian regulation than in the direct modulation of cognitive processes, while evidence concerning TMAO, PAGln, GABA-related compounds, and phenolic metabolites remains limited and heterogeneous. Further longitudinal and interventional human studies are needed to determine causality and clinical relevance. The principal associations between the microbiota-derived metabolites discussed in this section and circadian, sleep-related, and cognitive outcomes are summarized in Table 1.

**TABLE 1:** Microbiota-derived metabolites: key circadian, sleep-related, and brain-related findings.

Group	Examples	Circadian / sleep	Brain / cognition
SCFAs	Acetate; propionate; butyrate	Day–night variation; intestinal clock entrainment; peripheral clock shifts; disrupted plasma oscillations in shift workers; lower colonic butyrate after sleep deprivation	Barrier integrity; microglia; fiber deprivation–memory impairment; butyrate improved sleep-loss deficits; acetate/high-pectin linked to BDNF; observational human links
Tryptophan metabolites	Indole; IAA; IPA; tryptamine; 5-HTP	Diurnal tryptophan metabolism; altered gut barrier rhythms after microbial depletion; melatonin regulation; prolonged sleep in mice; reduced tryptophan and 5-HTP in circadian disruption	Barrier models; adult hippocampal neurogenesis; IPA in ASD/AD mouse models; cognitive improvement after tryptophan or 5-HTP supplementation; limited human ASD associations
Bile acids	Primary and secondary bile acids	Rhythmic bile acid metabolism; 24-h variation in healthy men; disrupted by sleep deprivation	Insomnia-associated signatures; altered profiles in Alzheimer cohorts; links with cognitive decline in aging and cognitive dysfunction in depression; taurodeoxycholic acid improved cognition in mice
TMAO	Trimethylamine N-oxide	Increased in blood and brain after chronic sleep restriction; exogenous TMAO worsened sleep-loss deficits in mice	Inverse association with memory/fluid cognition in one cohort; no overall association in Rotterdam Study; impaired memory-related performance in animal models; higher in mild cognitive impairment
PAGln	Phenylacetylglutamine	No direct rhythmicity data highlighted	Higher plasma PAGln associated with post-stroke cognitive impairment
GABA-related signaling	GABA-producing lactobacilli; *Bifidobacterium* strains	*B. breve* CCFM1025 attenuated circadian disturbance in sleep-deprived mice	Higher intestinal GABA and reduced anxiety-like behavior in mice; lower cognitive reactivity to sad mood in humans; improved novel object recognition in sleep-deprived mice
Phenolic metabolites	Phenolic metabolites; urolithin A	Protection against sleep-deprivation-induced cognitive impairment; BMAL1/PER2 modulation; attenuation of inflammation-related circadian dysregulation	Better frontal cognitive performance in older adults; reduced age-related memory impairment and inflammation in aged mice

5-HTP, 5-hydroxytryptophan; AD, Alzheimer’s disease; ASD, autism spectrum disorder; *B. breve*, *Bifidobacterium breve*; BDNF, brain-derived neurotrophic factor; BMAL1, brain and muscle ARNT-like 1; GABA, gamma-aminobutyric acid; IAA, indole-3-acetic acid; IPA, indole-3-propionic acid; PAGln, phenylacetylglutamine; PER2, period circadian regulator 2; SCFAs, short-chain fatty acids; TMAO, trimethylamine N-oxide.

## 5 Integrated axis (microbiota-circadian-cognition)

While the relationships between gut microbiota, circadian rhythms, and cognitive function have been increasingly studied in isolation, their integration into a unified, multi-level system remains less well defined. Emerging evidence suggests that these domains do not operate independently but rather form a dynamic and interconnected axis, in which temporal organization, microbial activity, and brain function are closely aligned. Understanding this integrated framework is essential for interpreting how disruptions in one domain may propagate across systems and ultimately influence cognitive outcomes.

### 5.1 Circadian modulation of cognitive function

Cognitive performance is not constant throughout the day but follows a distinct circadian pattern shaped by both endogenous biological rhythms and external behavioral cues. Processes such as attention, working memory, executive function, and decision-making exhibit time-of-day variation, reflecting the influence of the central circadian clock as well as peripheral oscillators across the body.

At the neurobiological level, circadian rhythms exert a broad regulatory influence on multiple systems underlying cognitive function. These include rhythmic modulation of neurotransmitter activity (particularly dopaminergic and serotonergic signaling involved in attention and executive control) (Kim et al., 2017), time-of-day variation in synaptic plasticity processes that support learning and memory consolidation (Frank, 2016), oscillations in hormonal systems such as cortisol that affect arousal and working memory performance (Androulakis, 2021), as well as circadian regulation of brain energy metabolism, which determines neuronal efficiency and cortical processing capacity (Maury, 2019). Collectively, these mechanisms contribute to diurnal fluctuations in cortical excitability and network-level communication, thereby shaping cognitive performance across the day.

Circadian misalignment – such as that experienced during shift work, jet lag, or irregular sleep–wake schedules – is associated with reduced attention, slower reaction times, impaired working memory, and decreased cognitive flexibility (Chellappa et al., 2019). Importantly, these findings support an independent contribution of circadian dysregulation to brain function, as performance deficits emerge even in controlled protocols where sleep duration and total time awake are held constant (Chellappa et al., 2018). Meta-analytic evidence further indicates that sustained attention, working memory, and executive control are significantly impaired during the biological night, with pooled effect estimates remaining significant after adjustment for sleep duration and prior wakefulness (Lim and Dinges, 2010; Short et al., 2015).

Early-life sleep disruption represents a particularly consequential form of circadian misalignment. Chronic sleep restriction or fragmentation in childhood is associated with impairments in attention, working memory, and executive functioning, as well as altered emotional regulation and increased impulsivity, with emerging evidence suggesting that these effects may persist beyond childhood by altering the developmental trajectories of prefrontal and limbic systems (Spruyt, 2019). Evening exposure to blue-wavelength light suppresses melatonin secretion and delays circadian phase, leading to impaired sleep quality and next-day deficits in vigilance and sustained attention - effects that appear secondary to circadian disruption rather than direct neural processing (Silvani et al., 2022; Cajochen et al., 2011; Chellappa et al., 2013).

Taken together, these findings support a fundamental role of circadian rhythms in cognitive function. Critically, many of the neurobiological systems through which circadian timing shapes cognition – including serotonergic and dopaminergic signaling, HPA axis rhythmicity, and synaptic plasticity – are also influenced by gut microbiota activity. This convergence positions the microbiota as a potential participant in the circuits that determine daily cognitive performance, rather than merely a passive correlate of circadian function.

### 5.2 Microbiota as a temporal modulator of brain function

Beyond the well-documented effects of circadian rhythms on cognition, the gut microbiota itself undergoes pronounced diurnal changes in both composition and metabolic output. These oscillations are closely coupled to host feeding–fasting cycles, suggesting that the microbiota may serve as an additional, temporally organized layer through which circadian signals reach the brain. Microbial metabolite production – including short-chain fatty acids and tryptophan-related compounds – is temporally organized in alignment with host circadian timing and feeding patterns. Disruption of this organization through irregular feeding, sleep deprivation, or light exposure may lead to desynchronization of microbial signaling from host biological timing, potentially impairing downstream brain function (Thaiss et al., 2014; Voigt et al., 2014). Experimental evidence supports this model. In animal studies, circadian disruption induces shifts in microbial composition alongside alterations in metabolite production, which are associated with increased systemic inflammation, impaired gut barrier integrity, and activation of neuroimmune pathways (Li et al., 2021; Eum et al., 2023). In a landmark study by Wang et al. (2021), transplantation of gut microbiota from sleep-deprived donors into germ-free recipient mice reproduced neuroinflammatory changes and deficits in learning and memory. This effect was mediated via activation of the Toll-like receptor 4/nuclear factor-κB (TLR4/NF-κB) inflammatory signaling pathway and increased production of pro-inflammatory cytokines, including tumor necrosis factor (TNF-α), IL-1β, and IL-6 (Wang et al., 2021).

Complementary findings demonstrated that chronic sleep deprivation-induced dysbiosis activates the NOD-, LRR- and pyrin domain-containing protein 3 (NLRP3) inflammasome both peripherally and centrally, contributing to neuroinflammation and cognitive dysfunction. Crucially, germ-free mice that had not received any microbiota transplant did not exhibit these cognitive impairments following sleep deprivation, indicating that the microbiota was necessary for these effects in this experimental model (Zhao et al., 2023).

Importantly, the timing of microbial activity may be as consequential as its overall composition. Time-of-day variation in SCFA production has been linked to modulation of histone deacetylase activity and circadian gene expression (Kuang et al., 2019), while oscillations in metabolite availability appear to regulate synaptic plasticity, microglial activation, and neurotransmitter balance (Leone et al., 2015; Dalile et al., 2019). When microbial signals occur at inappropriate circadian times, their physiological effects may be altered or maladaptive (Voigt et al., 2014), which may explain why circadian disruption is associated with cognitive impairment even in the absence of major changes in overall microbial diversity (Wang X. et al., 2023).

Taken together, these findings support a model in which the gut microbiota may act as a temporally organized interface between circadian regulation and brain function, linking disruptions in biological timing with neurophysiological and cognitive consequences.

### 5.3 Integrated mechanistic pathways linking microbiota, circadian rhythms, and cognition

The microbiota–circadian–cognition axis operates through at least three overlapping and mutually reinforcing pathways – neuroimmune signaling, metabolic-epigenetic regulation, and neuroendocrine crosstalk – each exhibiting circadian organization (Scheer et al., 2009; Cryan et al., 2019). One of the central components of this integration is the neuroimmune pathway. Circadian rhythms tightly regulate immune function, including cytokine production and inflammatory responses, which themselves exhibit diurnal variation (Scheiermann et al., 2013). Disruption of microbial rhythmicity has been associated with increased intestinal permeability and translocation of pro-inflammatory signals, which may contribute to systemic inflammation and activation of neuroimmune pathways (Thaiss et al., 2016). Elevated levels of cytokines such as IL-6 and TNF-α can influence sleep architecture, synaptic plasticity, and cognitive processes, including memory and attention. Importantly, these effects are not static but depend on circadian timing, as immune responses vary across the day–night cycle. Thus, misalignment between microbial activity and host circadian phase may amplify inflammatory signaling and contribute to cognitive dysfunction (Scheiermann et al., 2013). The metabolic-epigenetic pathway operates primarily through microbiota-derived metabolites. SCFAs and tryptophan metabolites modulate gene expression, neuronal activity, and barrier integrity (Dalile et al., 2019), and their circadian production patterns allow them to influence host clock gene expression through HDAC inhibition (Kuang et al., 2019). In the brain, these mechanisms affect synaptic plasticity, neurogenesis, and microglial function (Erny et al., 2015), with phase-specific metabolite exposure potentially producing differential physiological outcomes.

The neuroendocrine pathway provides an additional layer of integration. Hormonal systems central to circadian regulation, including cortisol and melatonin, are influenced by both host circadian clocks and microbial signals (Foster et al., 2017). The gut microbiota has been implicated in the regulation of hypothalamic-pituitary-adrenal (HPA) axis rhythmicity (Sudo et al., 2004), while microbial metabolites derived from tryptophan contribute to melatonin synthesis and sleep regulation (Jagannath et al., 2013). These interactions establish a bidirectional relationship in which circadian hormones shape microbial ecology, and microbial activity feeds back onto endocrine signaling (Cryan et al., 2019). Given the well-established role of cortisol and melatonin in modulating arousal, sleep, and cognitive function, this pathway represents a critical link between microbiota dynamics and brain performance.

These three pathways are synchronized by environmental cues – light exposure and feeding behavior – that entrain both host and microbial rhythms across the 24-h cycle (Zarrinpar et al., 2014; Leone et al., 2015). Disruption at one level can propagate across the system, and the resulting loss of temporal coordination may represent the one mechanism contributing to cognitive impairment associated with circadian dysregulation (Voigt et al., 2014).

**FIGURE 2:**
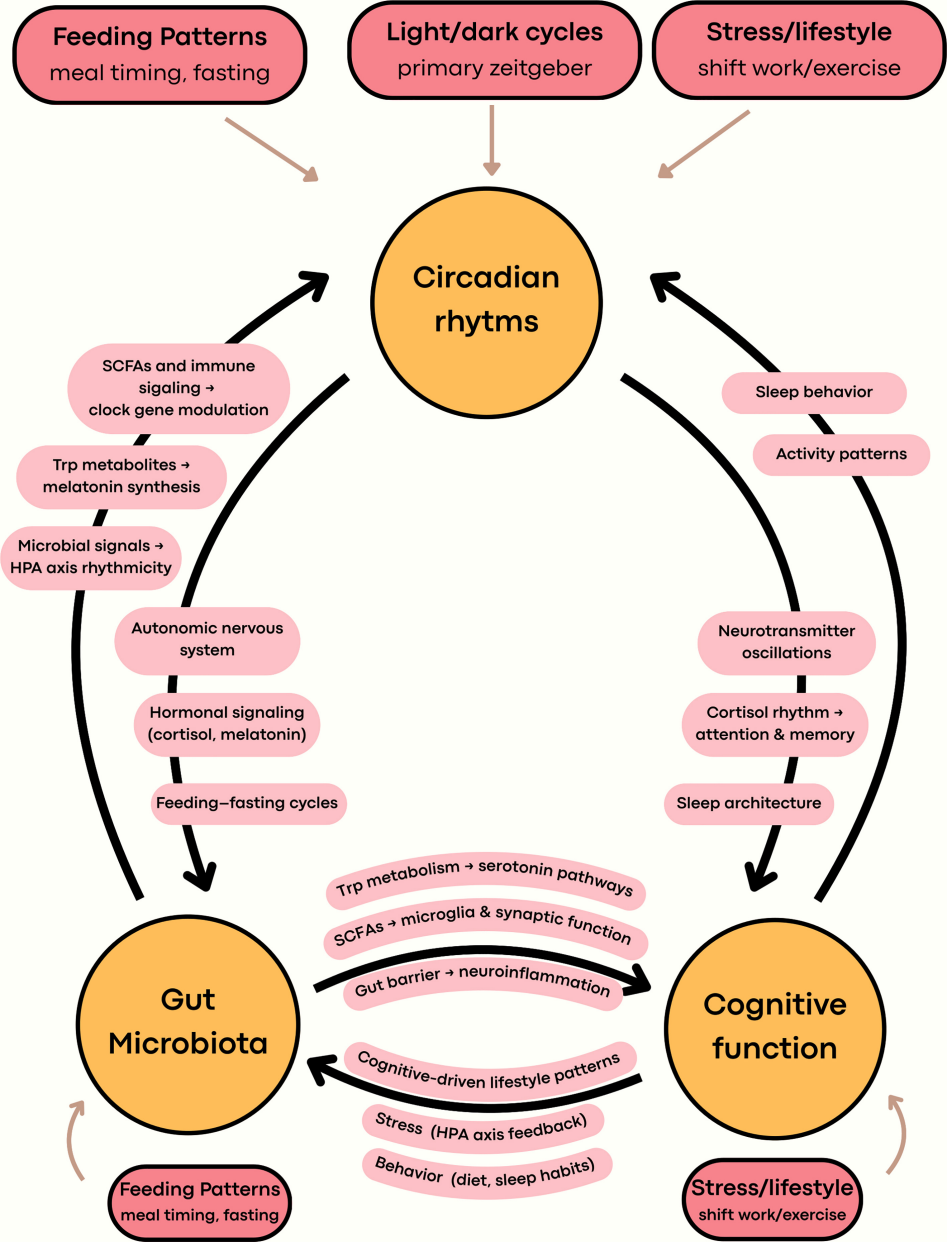
The microbiota–circadian–cognition axis: environmental modulators, bidirectional interactions, and key mediators. HPA, hypothalamic-pituitary-adrenal; SCFAs, short-chain fatty acids; Trp metabolites, tryptophan metabolites.

Overall, the microbiota–circadian–cognition axis can be understood as a temporally integrated network in which microbial signals, host biological clocks, and brain function are tightly interconnected. Within this framework, timing emerges as a fundamental determinant of system-wide coherence, highlighting the importance of considering not only the components of the axis but also their synchronization in shaping cognitive outcomes. The key domains of this axis, together with their principal mediators and environmental modulators, are summarized in Figure 2.

This integrated framework has potential implications for how interventions targeting the gut microbiota or circadian alignment might be designed and timed. Recognizing that microbial signals, host biological rhythms, and brain function form a mutually dependent system, the following section examines practical strategies that may leverage this axis to support cognitive health.

## 6 Practical application

The growing recognition of interactions between the gut microbiota, circadian rhythms, and brain function provides a rationale for investigating strategies that may support cognitive health. While current evidence is largely observational, some approaches may have potential relevance for clinical practice.

### 6.1 Chrononutrition and meal timing

One of the most accessible candidate intervention targets within this axis is the timing of food intake. Feeding–fasting cycles act as key synchronizers of both host circadian rhythms and microbial oscillations. In human observational studies, circadian misalignment – particularly in shift workers – has been associated with reduced microbial diversity and increased abundance of pro-inflammatory taxa, changes linked to metabolic dysfunction and gastrointestinal symptoms that may indirectly affect brain function through systemic pathways (Grasa-Ciria et al., 2025).

Restricting daily food intake to a 6–10-h window (corresponding to 14–18 h of fasting) is one of the most commonly applied and effective approaches, with meta-analytic evidence showing improvements in metabolic outcomes and emerging data suggesting favorable effects on gut microbiota composition and circadian regulation (Rovira-Llopis et al., 2024).

The timing of food intake also appears relevant independently of window duration, with earlier eating windows showing more favorable metabolic and circadian outcomes than late-day consumption (Adafer et al., 2020).

### 6.2 Sleep and circadian alignment

Sleep and circadian alignment represent critical components of the microbiota–circadian–cognition axis with clear clinical relevance for cognitive function. Circadian disruption, such as night shift work, has been associated with microbiota alterations and impairments in cognitive functions, as well as increased risk of neurocognitive and metabolic disorders (McHill et al., 2014; Moosavi et al., 2025). Epidemiological data indicate that a substantial proportion of shift workers report gastrointestinal symptoms potentially associated with altered microbial composition (Rogers et al., 2021), while experimental studies demonstrate that transitions between shift types can alter microbiota composition within weeks (Grasa-Ciria et al., 2025). From a cognitive perspective, circadian misalignment can impair attention, executive function, and working memory (Moosavi et al., 2025), and microbiota-related metabolic and neuroendocrine changes may contribute to these effects. Practical strategies to mitigate these effects include maintaining consistent sleep schedules, morning bright light exposure to reinforce circadian phase, light avoidance in the post-shift period, short restorative naps, and gradual forward-rotating shift patterns (moving from day shift to evening shift to night shift) (James et al., 2017).

### 6.3 Microbiota-targeted interventions

Dietary strategies targeting the gut microbiota represent a promising but still developing area of intervention, particularly in the context of the microbiota–circadian–cognition axis. Beyond general dietary patterns, increasing attention has been directed toward the use of probiotics and prebiotics as targeted tools to modulate gut microbial composition and function. Preclinical data suggest that probiotics may help restore circadian rhythmicity following disruption, potentially through modulation of microbial metabolites and host signaling pathways (Liang et al., 2015; Thaiss et al., 2016).

Evidence from randomized controlled trials and meta-analyses indicates that probiotic supplementation may lead to modest improvements in cognitive performance – including memory, processing speed, and executive function – particularly in older adults and individuals with cognitive impairment (Calzada-Gonzales et al., 2025; Guo et al., 2026). Specific strains such as *Bifidobacterium breve* have shown cognitive benefits in placebo-controlled trials, and longer-term interventions (≥12 weeks) appear associated with more pronounced effects (Xiao et al., 2020).

Prebiotic supplementation has also shown preliminary effects on stress-related cognitive processing, including reductions in waking cortisol (Schmidt et al., 2015). Regarding sleep and circadian outcomes, probiotic interventions have produced inconsistent results in humans, with some trials reporting improvements in sleep onset latency and daytime fatigue, while others show no significant effect on overall sleep quality (Chu et al., 2023; Liu et al., 2025). Fecal microbiota transplantation (FMT) has shown preliminary promise in chronic insomnia, with one real-world study reporting reductions in insomnia severity alongside shifts in microbiota composition (Fang et al., 2023), though controlled trials in cognitive outcomes are lacking.

Taken together, microbiota-targeted interventions show preliminary promise for both cognitive function and circadian-related outcomes, though causal mechanisms remain to be established. The practical strategies discussed in this section, along with their proposed mechanisms and the current, largely preliminary, state of human evidence, are summarized in Table 2.

**TABLE 2:** Practical strategies targeting the microbiota–circadian–cognition axis.

Strategy	Proposed mechanism	Key evidence	Evidence level
Time-restricted eating (8–10 h window)	Entrains host and microbial circadian rhythms via feeding–fasting cycles; reduces pro-inflammatory taxa	Meta-analyses: improved metabolic outcomes; observational data on microbiota shifts in shift workers	Moderate
Earlier eating window (before 16:00)	Better alignment with cortisol peak and metabolic circadian phase; reduces late-night microbial dysregulation	Observational studies; more favorable metabolic and circadian outcomes vs. late eating	Preliminary
Consistent sleep schedule	Maintains SCN entrainment; preserves microbial diversity and SCFA production rhythmicity	Epidemiological data linking sleep regularity to alpha-diversity and cognitive performance	Moderate
Morning bright light exposure	Reinforces SCN circadian phase; suppresses residual melatonin; aligns peripheral clocks	Controlled studies in shift workers; systematic reviews on blue light and circadian phase	Good
Probiotic supplementation (e.g., *Lactobacillus*, *Bifidobacterium*)	Modulates microbiota composition; influences tryptophan and SCFA signaling; may improve sleep onset	RCTs and meta-analyses; modest effects on sleep quality, stress, and cognitive performance	Moderate
Prebiotic supplementation (e.g., GOS)	Increases SCFA-producing taxa; reduces waking cortisol; attenuates stress-related cognitive bias	Single placebo-controlled RCT (Schmidt et al., 2015); limited replication	Preliminary
Fecal microbiota transplantation (FMT)	Restores microbial diversity; shifts bile acid and SCFA profiles; reduces insomnia severity	Real-world study (Fang et al., 2023); no RCTs in cognitive outcomes	Preliminary

GOS, galactooligosaccharides; RCT, randomized controlled trial; SCN, suprachiasmatic nucleus; SCFA, short-chain fatty acid. Evidence levels - Good: consistent findings across multiple controlled studies or meta-analyses; Moderate: RCT or meta-analytic evidence with some limitations; Preliminary: single studies, animal models, or observational data only.

## 7 Discussion

### 7.1 Conceptual considerations and current gaps

Despite increasing recognition of the microbiota–circadian–cognition axis, the current evidence base remains fragmented. Most studies focus on individual components or pairwise interactions without capturing the full system-level complexity, and the extent to which these components interact simultaneously in humans remains incompletely understood.

Two limitations are particularly consequential. First, the insufficient incorporation of temporal factors into study design – many studies assess microbiota composition or cognitive function at single time points, without accounting for diurnal variation or the timing of interventions and sample collection. Second, causal relationships remain difficult to establish: experimental models provide mechanistic insights, although their translational relevance to human physiology remains uncertain, while human studies are often observational or of short duration, making it challenging to disentangle cause-and-effect relationships within this complex axis.

The findings reviewed here are therefore best interpreted as supporting the biological plausibility of the axis and highlighting potential mechanistic pathways, rather than confirming causal relationships or the efficacy of specific interventions.

### 7.2 Future directions

Progress in this field would benefit from studies that integrate the temporal dimension more explicitly. Well-designed human trials simultaneously assessing microbiota composition, circadian parameters, and cognitive outcomes are needed, alongside standardization of microbiome sequencing approaches and cognitive assessment tools to improve comparability across studies. Longitudinal and interventional studies with larger sample sizes are essential to determine whether microbiota modulation can lead to sustained cognitive improvements, with particular attention to the timing of interventions relative to the host’s circadian phase.

One underexplored direction concerns chronotype as a moderating variable. Given the reported associations of chronotype with both microbiota composition and cognitive performance, future studies should consider stratifying participants by chronotype – this may help explain some of the inconsistencies across studies and support the development of more personalized intervention approaches.

Sex and biological differences represent another important and currently underexplored dimension. Circadian rhythms, gut microbiota composition, and cognitive trajectories are all known to differ between sexes, yet the majority of existing studies do not stratify by sex or account for hormonal influences such as the menstrual cycle or menopause. Future research should explicitly incorporate sex as a biological variable to better understand how these factors modulate the microbiota–circadian–cognition axis and to ensure that intervention strategies are appropriately tailored.

Aging represents a further context warranting dedicated investigation. Both circadian rhythm robustness and microbial diversity decline with age, and their concurrent deterioration may amplify vulnerability within the microbiota–circadian–cognition axis. Future studies should therefore include older adults as a priority population, given that age-related changes in both systems may modify the magnitude and direction of observed effects.

At the other end of the lifespan, children and adolescents represent an equally important but understudied group. Both the gut microbiota and circadian system undergo substantial maturation during development, and early disruptions – such as irregular sleep schedules, highly processed diets, or excessive evening light exposure – may have disproportionate and potentially lasting effects on the developing microbiota–circadian–cognition axis. Longitudinal studies tracking microbiota composition, circadian parameters, and cognitive outcomes across childhood and adolescence are needed to better understand how early-life exposures shape this axis and whether developmental windows of heightened sensitivity exist.

More broadly, the field would benefit from greater attention to individual variability in intervention responses. Baseline microbiota composition, genetic polymorphisms in clock genes, lifestyle factors such as physical activity and diet quality, and comorbid conditions all likely influence the magnitude and direction of effects observed in microbiota- or circadian-targeted interventions. Precision approaches that integrate these variables – for example through multi-omics profiling at baseline – may help identify subgroups most likely to benefit and explain inconsistent findings across trials.

Finally, multi-omics techniques and advanced analytical methods may better characterize the interactions between microbial metabolites, circadian regulation, and cognition.

Regarding practical strategies, their efficacy may vary according to individual chronotype, baseline microbial composition, and intervention timing – variables that have not been consistently controlled in existing trials. Future studies should therefore not only assess whether such interventions work, but also when they work best.

### 7.3 Study limitations

The main limitations of this review reflect both the nature of the available evidence and the methodology applied. The evidence base is highly heterogeneous, encompassing preclinical models, observational studies, and small clinical trials with variable assessment methods. Animal studies provide mechanistic insights but uncertain translational relevance, while human studies are largely associative with inconsistently controlled confounders, including diet, sleep patterns, and inter-individual microbiota variability.

As a narrative review, this work does not follow a systematic methodology, and study selection may be subject to selection bias. The temporal dimension is insufficiently addressed in much of the existing literature – many studies do not account for the timing of sample collection or interventions despite evidence of diurnal fluctuations in host physiology and microbial activity, which may obscure or underestimate the role of rhythmicity within the axis. The absence of a formal risk-of-bias evaluation further limits the strength of conclusions that can be drawn.

These findings should therefore be read as a qualitative synthesis aimed at integrating current knowledge and identifying research gaps, rather than a quantitative assessment of evidence certainty.
